# Can Ultrasound Elastography Be Used as an Objective Measure in Tonsillectomy Indications?

**DOI:** 10.3390/jcm15145413

**Published:** 2026-07-10

**Authors:** Feride Fatma Görgülü, Orhan Görgülü

**Affiliations:** 1 Department of Radiology, Adana Health Practice and Research Center, University of Health Sciences, Adana 01330, Turkey; 2 Department of Otorhinolaryngology, Adana Health Practice and Research Center, University of Health Sciences, Adana 01330, Turkey

**Keywords:** tonsillitis, adenotonsillar hypertrophy, tonsillectomy, elasticity imaging techniques, shear wave elastography, ultrasonography, diagnostic imaging

## Abstract

**Objectives**: The objective of this study was to evaluate the diagnostic performance of the strain ratio (SR) and shear wave elastography (SWE) in distinguishing normal tonsils from those affected by adenotonsillar hypertrophy (ATH) and chronic tonsillitis (CT), and to assess their correlation and clinical applicability. **Methods**: This study consisted of 96 participants, which included 31 controls, 55 with ATH, and 10 with CT. The SR was measured, and SWE was performed bilaterally. After Shapiro–Wilk testing confirmed non-normality, groups were compared with the Kruskal–Wallis test and Dunn–Bonferroni post hoc analysis; differences in sex distribution were determined with the chi-square test; and the SR–SWE correlation was assessed with Spearman’s coefficient. Diagnostic accuracy was assessed via ROC analysis (AUC, sensitivity, and specificity). **Results**: SWE and submandibular gland-based SR values were consistently higher in the ATH and CT groups than in controls, whereas the tongue-based SR showed less consistent differences. SWE achieved excellent diagnostic accuracy (AUC = 0.918 right, 0.892 left), outperforming the SR (highest AUC = 0.844). SWE achieved the highest sensitivity, and right-sided SWE achieved the highest specificity. SWE results correlated positively with the submandibular gland-based SR, whereas the tongue-based SR showed no consistent correlation. No sex-related differences were found (*p* = 0.587). **Conclusions**: Elastography effectively differentiates normal from pathological tonsillar tissue, with SWE providing superior diagnostic accuracy. As a non-invasive adjunct to conventional ultrasonography, SWE may help assess tonsillar disease severity, guide clinical management, and avoid unnecessary surgery.

## 1. Introduction

Tonsillectomy is one of the most commonly performed operations in otolaryngology, particularly in children and adolescents. This surgery is primarily indicated for recurrent or chronic tonsillitis (CT) and for adenotonsillar hypertrophy (ATH), which leads to upper airway obstruction or sleep-disordered breathing [1,2]. Although this is a longstanding and widely used procedure, the decision to operate still relies largely on subjective information, such as the reported frequency and severity of infections and their impact on quality of life [3]. The criteria are commonly applied based on several parameters such as clinical history, physical examination results, or patient or parental reports of how often tonsillitis occurs or how obstructed the airway appears [4]. However, these parameters are vulnerable to recall bias, interobserver variance, and a lack of standardized objective measures. As a result, there remains a clear need for quantitative and reproducible diagnostic tools that could provide an objective basis for indications of tonsillectomy.

The palatine tonsils are lymphoid glands that form part of Waldeyer’s ring and contribute to mucosal immune defense. Chronic infection or hypertrophy can produce structural and functional changes within the tissue, including fibrosis, vascularization changes, and increased cellular density [5]. Such tissue-level remodeling possibly affects the mechanical characteristics of the tonsils, particularly their stiffness/elasticity. Traditional imaging modalities like computed tomography or magnetic resonance imaging (MRI) have occasionally been used to assess asymmetry, suspected malignancy, or deep neck infection, but not to assess functional tissue characteristics. Preoperative imaging has therefore never become part of routine tonsillectomy decision-making, largely because these modalities are not sensitive enough to detect subtle soft-tissue abnormalities [6]. Thus, a technique capable of quantifying tonsillar tissue properties could have significant implications for clinical management.

Over the past two decades, ultrasound elastography has emerged as a non-invasive imaging technique that measures the level of tissue stiffness, an indicator of the mechanical and structural state of tissue [7,8]. Tissue stiffness is estimated from the degree of deformation produced during elastography by applied pressure or by propagating shear waves [9]. Two main approaches are used, namely strain elastography and shear wave elastography (SWE) [10]. Strain elastography measures relative tissue stiffness by comparing the deformation of a region of interest with that of a reference tissue; it is usually reported as a strain ratio (SR), where higher values indicate stiffer tissue [11]. SWE uses acoustic radiation force to produce shear waves and measures their propagation speed, which is proportional to tissue elasticity and is expressed in kilopascals (kPa) [12]. The two techniques provide complementary information. Strain elastography allows relative comparison between structures, whereas SWE yields absolute quantitative values. Because the palatine tonsils are superficial and readily accessible to high-frequency ultrasound, they are well suited to elastographic evaluation [13].

Previous studies have shown that chronic inflammation and fibrosis tend to increase tissue stiffness on elastography, and persistent inflammation, lymphoid hyperplasia, and interstitial fibrosis might likewise alter tissue elasticity in CT and ATH [6]. Nevertheless, relatively few studies have investigated elastography of the palatine tonsils specifically [14]. The available evidence is largely limited to normative shear wave elastography values in healthy children and adolescents [15,16], with little data on how these values change in tonsillar disease. Most early research has concentrated on differentiating benign from malignant cervical lymph nodes, on evaluating the tongue and salivary glands, or on measuring muscle stiffness. However, the use of elastography in routine tonsillar examination, especially in support of tonsillectomy decisions, has not been explored in depth. Therefore, the value of elastography in providing an objective imaging biomarker for tonsillar disease remains to be established.

Elastography may also be useful in addressing one of the main limitations of current tonsillectomy decision-making, which is the absence of measurable and reproducible imaging parameters. If quantitative elastographic values differ consistently among patients with tonsillar disease and healthy individuals, these values could complement existing clinical criteria, improve diagnostic accuracy, reduce unnecessary surgery, and provide objective measures for follow-up and treatment planning. On this basis, this study was designed to compare ultrasound elastography parameters, SR and SWE measurements, between children scheduled for tonsillectomy and healthy controls. We hypothesized that ultrasound elastography can provide an objective, quantitative measure to support clinical indications for tonsillectomy, and we further aimed to determine which parameter, SR or SWE measurements, best discriminates pathological from normal tonsillar tissue.

## 2. Materials and Methods

### 2.1. Study Design and Ethical Considerations

This prospective cross-sectional study was conducted in the outpatient clinics of the Department of Otorhinolaryngology and Radiology at Adana City Hospital. The study protocol was approved by the Clinical Research Ethics Committee of Adana City Hospital (№ 314/07-11-18, issued on 7 November 2018), and written informed consent was obtained from each participant or their legal guardian prior to enrollment. The study was conducted in accordance with the principles of the Declaration of Helsinki. Participation was entirely voluntary, and confidentiality of patient information was strictly maintained. All procedures were non-invasive, and no adverse effects or complications were observed during or after imaging.

### 2.2. Participants

The participants were allocated to three groups. The ATH group comprised patients with a clinical diagnosis of tonsillar hypertrophy who met the standard indications for surgery. The CT group included patients with recurrent or chronic tonsillar infection that necessitated tonsillectomy. The participants in the ATH and CT groups underwent surgery after our measurements, receiving either adenotonsillectomy or just tonsillectomy. The control group consisted of healthy volunteers with no history of recurrent throat infection, no clinical evidence of tonsillar enlargement, and no abnormalities on oropharyngeal examination. Eligible participants were aged between 3 and 17 years, had a clinical diagnosis of ATH or CT established according to standard ENT criteria, were scheduled for surgery, and were free of acute infection during the examination. Participants were excluded if they had a history of prior tonsillectomy or other oropharyngeal surgery; had a suspected peritonsillar abscess, malignancy, or systemic inflammatory disease; had a poor acoustic window; or inadequately cooperated with the ultrasonography procedure.

### 2.3. Procedures—Ultrasound and Elastography Technique

All examinations were conducted using a high-frequency linear and convex transducer on a single ultrasound system. Participants were positioned supine with a slight extension of the neck and with their head turned in the opposite direction to the investigated side to optimize acoustic coupling of the submandibular region. Patients also were asked to hold their breath and not swallow during the examination in order to minimize movement of the tonsil, the tongue, and the submandibular gland. The palatine tonsils were evaluated bilaterally in every participant. The radiologist took approximately 5–7 min to perform the examination and evaluation for each patient.

#### 2.3.1. Strain Elastography

**Figure 1:**
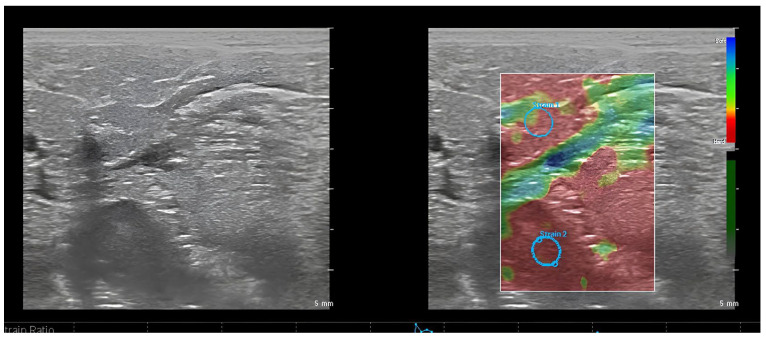
Measurement of the SR in the right-side tonsil and submandibular gland (the upper ROI represents the submandibular gland, while the lower ROI indicates the tonsil).

**Figure 2:**
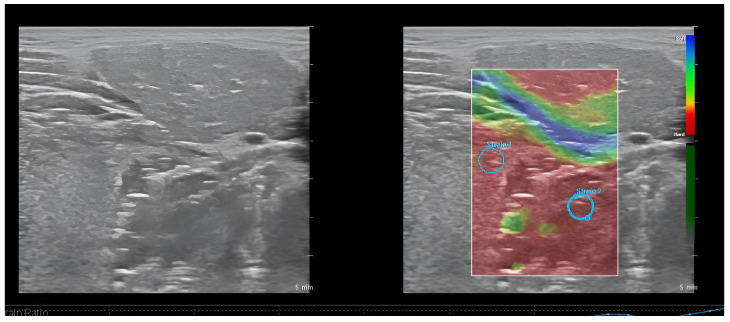
Measurement of the SR in the left-side tonsil and tongue tissue (the upper ROI represents the tongue tissue, while the lower ROI indicates the tonsil).

Strain elastography was performed using a high-resolution Doppler ultrasound system (Philips EPIQ 7, Philips Healthcare, Bothell, WA, USA) equipped with a 5–18 MHz linear probe (Philips L18-5). Strain images were generated by applying gentle, repetitive manual compression. Strong initial compression was avoided so as not to increase the probability of false-negative results. For each participant, the SR of the tonsil was calculated relative to two reference tissues on the same side, namely the tongue muscle and the submandibular gland, which yielded four measurements per participant. The radiologist selected rounded regions of interest (ROIs), including the tonsil and the tongue or the submandibular gland, as references in order to provide a semi-quantitative analysis (3 × 3 mm circular ROI box). These ROIs were the right tonsil-to-tongue (SR1), left tonsil-to-tongue (SR2), right tonsil-to-submandibular gland (SR3), and left tonsil-to-submandibular gland (SR4) (Figure 1 and Figure 2). The system automatically computed the SRs from the strain of the target and reference tissues, with higher values indicating greater tonsillar stiffness relative to the reference tissue.

#### 2.3.2. Shear Wave Elastography (SWE)

**Figure 3:**
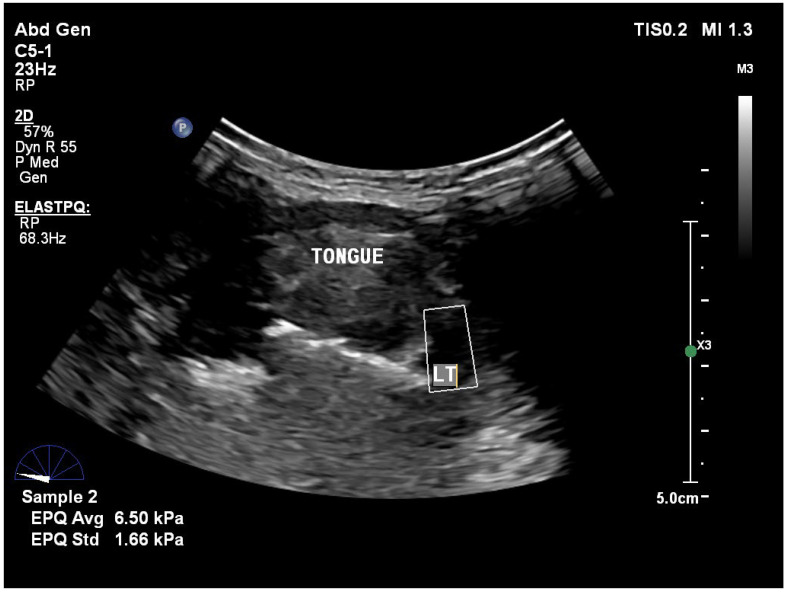
SWE evaluation of the left palatine tonsil.

Following strain elastography, SWE was performed bilaterally on all tonsils via a transcervical approach using the same system (Philips EPIQ 7 Philips Health Care, Bothell, WA, USA) with a C5-1 16 MHz high-resolution convex probe using ELAST PQ software. A region of interest was placed within the tonsillar parenchyma, avoiding visible vessels and cystic lesions (Figure 3). The measurements are presented in kilopascals (kPa). The SWE elasticity values of the tonsils were measured three times using three different ROIs of 5 × 10 mm from different areas for each tonsil by the same observer, and the average of these measurements was recorded as the final data. All images were stored for subsequent review, and all measurements were performed by the same operator to minimize interobserver variability.

All examinations were performed during a single clinic session by an experienced head and neck radiologist who was blinded to the clinical group assignment of the participants.

### 2.4. Statistical Analysis

The normality of continuous variables was assessed with the Shapiro–Wilk test. As the continuous variables did not follow a normal distribution, non-parametric methods were used throughout. Continuous variables were summarized as medians (interquartile ranges, IQRs) and categorical variables as frequencies and percentages. Between-group differences in continuous variables were assessed with the Kruskal–Wallis test, and significant results were followed up with pairwise comparisons using Dunn–Bonferroni correction. Differences in sex distribution among the groups were evaluated with the chi-square test. Correlations between SR and SWE measurements were assessed using the Spearman rank correlation coefficient. The diagnostic performance of each elastography parameter in discriminating patients with tonsillar disease (the combined ATH and CT groups) from healthy controls was evaluated by means of receiver operating characteristic (ROC) curve analysis. The area under the curve (AUC) was calculated, and the optimal cut-off value, sensitivity, and specificity were determined using the Youden index. All tests were two-tailed, and a *p*-value of <0.05 was considered statistically significant. All analyses were conducted using R (version 4.2.2).

## 3. Results

### 3.1. Demographic Characteristics

A total of 96 subjects were recruited in this study, comprising 55 patients with ATH, 10 patients with CT, and 31 healthy controls. The demographic characteristics of the study population are summarized in Table 1.

The median age was 8 (6–12) years in the ATH group, 14 (11.5–15) years in the CT group, and 8 (5–12) years in the control group. Age differed significantly among the groups (Kruskal–Wallis test, *p* = 0.003). Post hoc analysis demonstrated that patients with CT were significantly older than both patients with ATH (adjusted *p* = 0.005) and healthy controls (adjusted *p* = 0.004), whereas no significant age difference was observed between the ATH and control groups.

There was no significant difference in sex distribution among the groups (χ^2^ = 1.067, df = 2, *p* = 0.587). Female participants accounted for 43.6%, 60.0%, and 41.9% of the ATH, CT, and control groups, respectively.

### 3.2. Comparison of Elastography Parameters

Significant differences were observed among the three groups for all evaluated elastography parameters, including bilateral tonsil–tongue strain ratios, bilateral tonsil–submandibular gland strain ratios, and bilateral SWE measurements (all *p* < 0.001) (see Table 2).

**Figure 4:**
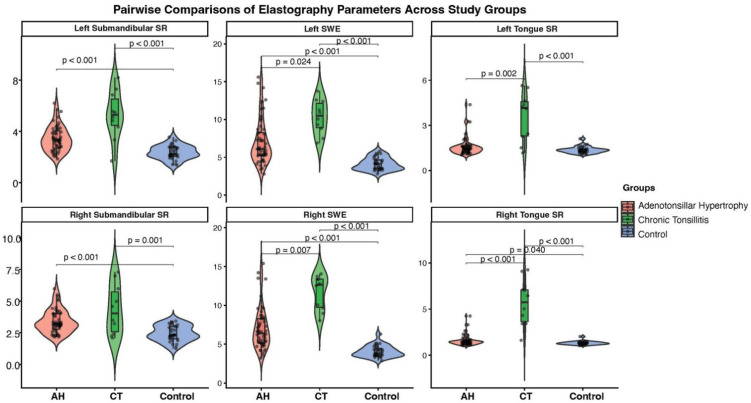
Post hoc pairwise comparisons of strain ratio (SR) and shear wave elastography (SWE) measurements among the adenotonsillar hypertrophy (AH), chronic tonsillitis (CT), and control groups. Violin plots depict the distributions of left and right tonsil-to-tongue SR, tonsil-to-submandibular gland SR, and tonsillar SWE values. Significant pairwise differences are indicated by *p*-values.

Post hoc Dunn–Bonferroni analyses demonstrated that chronic tonsillitis was associated with significantly higher bilateral SWE and tonsil–tongue strain ratio values compared with both ATH patients and controls. In contrast, bilateral tonsil–submandibular gland strain ratio values did not significantly differ between the CT and ATH groups. Compared with controls, patients with ATH exhibited significantly increased bilateral SWE values and bilateral tonsil–submandibular gland strain ratio values. For tonsil–tongue strain ratio measurements, only the right-sided parameter remained significantly different between ATH patients and controls. Comparisons of elastography parameters among the study groups are summarized in Figure 4.

### 3.3. Relationship Between Strain Ratio and SWE Measurements

In the whole cohort, significant positive correlations were identified between the strain ratio and SWE measurements. The strongest associations were observed between the right tonsil–submandibular gland strain ratio and right SWE measurements (ρ = 0.676, *p* < 0.001) and between the left tonsil–submandibular gland strain ratio and left SWE measurements (ρ = 0.606, *p* < 0.001).

**Figure 5:**
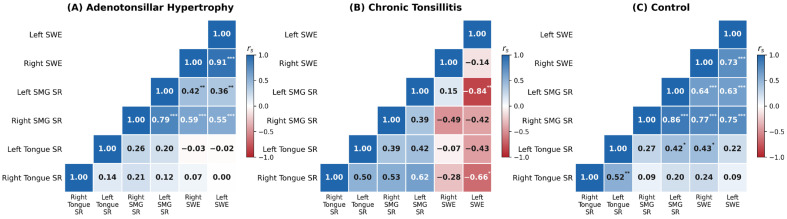
Spearman correlation matrices of elastography parameters in the (**A**) adenotonsillar hypertrophy, (**B**) chronic tonsillitis, and (**C**) control groups. Correlation coefficients (r_s_) are displayed within each cell, with the color intensity indicating the strength and direction of the association. Asterisks denote statistically significant correlations (* *p* < 0.05, ** *p* < 0.01, *** *p* < 0.001). SR, strain ratio; SMG, submandibular gland; SWE, shear wave elastography; r_s_, Spearman correlation coefficient.

Subgroup analyses revealed heterogeneous correlation patterns. In the ATH group, significant positive correlations were found between bilateral tonsil–submandibular gland strain ratios and corresponding SWE measurements. In the CT group, a significant correlation was observed only between the left tonsil–submandibular gland strain ratio and left SWE measurements. In controls, strong positive correlations were identified between bilateral tonsil–submandibular gland strain ratios and SWE measurements. In contrast, correlations between tonsil–tongue strain ratios and SWE values were generally weak and inconsistent across the subgroups. Correlation analyses are presented in Figure 5.

### 3.4. Diagnostic Performance of Elastography Parameters

Among all evaluated parameters, SWE values yielded the highest discriminative ability. Right-sided SWE achieved an AUC of 0.918 (95% CI, 0.864–0.973), followed closely by left-sided SWE (AUC 0.892; 95% CI, 0.830–0.955). At the Youden-derived cut-off of 5.15 kPa, right SWE reached a sensitivity of 78.5% and a specificity of 96.8%, while left SWE (cut-off 5.25 kPa) yielded the same sensitivity of 78.5% with a specificity of 87.1%. Both SWE parameters outperformed all strain ratio measurements in their overall discriminative ability.

**Figure 6:**
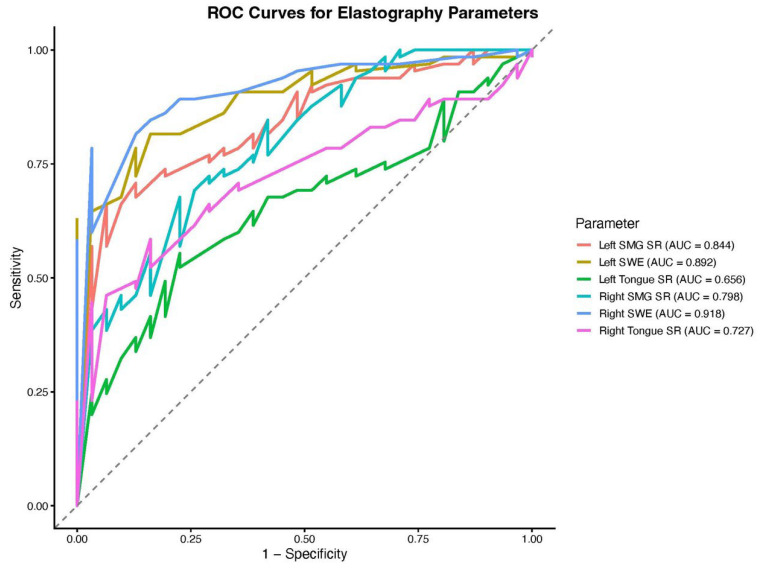
The receiver operating characteristic (ROC) curves of elastography parameters for differentiating the study groups. The dashed diagonal line represents the reference line for no discriminative ability (AUC = 0.50). ROC, receiver operating characteristic; AUC, area under the curve; SR, strain ratio; SWE, shear wave elastography; SMG, submandibular gland.

Among the strain ratio parameters, tonsil–submandibular gland ratios discriminated disease better than tonsil–tongue ratios. The left tonsil–submandibular gland strain ratio showed the highest accuracy among the strain-based measurements (AUC 0.844; 95% CI, 0.767–0.920; cut-off 3.08; sensitivity 66.2%, specificity 93.5%), followed by the right tonsil–submandibular gland ratio (AUC 0.798; 95% CI, 0.706–0.889). The tonsil–tongue ratios performed worst, with the left tonsil–tongue ratio showing the lowest accuracy overall (AUC 0.656; 95% CI, 0.546–0.766). The diagnostic performance of elastography parameters for differentiating patients with tonsillar disease from healthy controls is summarized in Table 3 and Figure 6.

## 4. Discussion

This study assessed whether SR and SWE measurements can objectively distinguish normal palatine tonsils from those affected by ATH and CT. All elastographic parameters differed significantly across the three groups, and diseased tonsils were consistently stiffer than those of controls. On ROC analysis, SWE showed the highest diagnostic accuracy, followed by the tonsil–submandibular gland SR. These findings indicate that elastography, and SWE in particular, has strong potential as a quantitative imaging tool for the assessment of tonsillar disease.

SWE provided the best diagnostic performance among the elastography methods. The median tonsillar stiffness was markedly higher in the disease groups than in controls, increasing progressively from controls to ATH and reaching its highest values in CT. The high discriminatory power of SWE confirms its value in separating normal from diseased tissue. This increase in stiffness most likely reflects the combined effect of lymphoid hyperplasia, persistent inflammation, and fibrotic remodeling [17] and is consistent with reports that SWE reflects fibrosis and inflammatory activity in other soft tissues such as the liver and lymph nodes [18]. A meta-analysis study confirmed its high accuracy for staging hepatic fibrosis [19]. Higher tonsillar stiffness in pediatric inflammatory disease has likewise been demonstrated with SWE in acute tonsillitis [14]. The strong bilateral correlation of SWE values in the ATH and control groups supports the largely symmetric nature of tonsillar involvement. Because SWE expresses stiffness in absolute, standardized units with limited operator dependence, it represents a quantitative complement to conventional ultrasonography. The absolute control values in our cohort were lower than those reported in earlier normative studies of healthy children [15,16], a difference most plausibly explained by variations in probe selection, measurement approaches, and patient ages, and one that underscores the need to interpret SWE thresholds within each technical setting.

Strain ratio elastography showed the same overall direction, with higher SR values in disease groups than in controls. This is in keeping with the greater tissue stiffness expected in tonsillar inflammation and hypertrophy [17], in which the entailed chronic immune stimulation leads to fibrosis, lymphoid hyperplasia, and stromal remodeling [20]. Elevated strain ratios in inflamed or hypertrophic tonsils have also been reported, reflecting increased collagen deposition and reduced tissue compliance [21]. Importantly, the choice of reference tissue strongly influenced performance. The submandibular gland-based SR discriminated disease considerably better than the tongue-based SR, and only the submandibular gland-based SR correlated consistently with SWE values, whereas the tongue-based SR showed negligible correlation. This indicates that the submandibular gland is a more stable internal reference than the tongue for tonsillar strain measurement. The gland shows consistent elastographic stiffness in healthy conditions, but its stiffness rises with structural changes such as post-radiation fibrosis [22], which supports its suitability as a reference tissue. The SR, nonetheless, remains semi-quantitative and operator-dependent and is therefore more susceptible to variability arising from probe pressure, patient motion, and limited oropharyngeal access, particularly in children [12]. Even so, the significant between-group differences indicate that the SR retains diagnostic value in settings where SWE is unavailable.

The elevated SR and SWE values in both ATH and CT can be explained by their underlying histopathology. ATH arises from chronic antigenic stimulation that drives lymphoid hyperplasia, stromal expansion, and fibrosis, all of which stiffen the tissue [23]. CT, in contrast, is characterized by repeated inflammatory injury leading to micro-fibrosis, collagen deposition, and scarring, structural changes that impede tissue deformation and raise elastographic values [24]. The same mechanism has been described in cervical lymph nodes and salivary glands, in which fibrosis and inflammation increase measured stiffness [25], and quantitative SWE has been shown to separate benign from pathological cervical nodes on this basis [26]. The particularly high stiffness of the CT group is consistent with cumulative fibrotic change; however, this group was also the smallest and its members were the oldest, so its values should be interpreted with caution.

Clinically, elastography offers a non-invasive, quantitative means of characterizing tonsillar disease, which is a distinction that is not always possible from clinical examination or tonsil size alone. Indeed, in children referred for adenotonsillectomy, clinical examinations and questionnaires are not sufficiently sensitive to identify obstructive sleep apnea, which shows the need for objective measures [27]. Given its high diagnostic accuracy, SWE may help identify patients who require surgical evaluation rather than conservative management. In our data, an optimal SWE cut-off separated normal from diseased tonsils and could serve as a practical clinical threshold, in accordance with the use of SWE stiffness cut-offs to flag lesions that require surgical evaluation in other tissues such as the thyroid [28]. It should be noted, however, that the sensitivity values in our cohort (55–79%) were considerably lower than the specificity (77–97%), particularly for SWE. This asymmetry suggests that elastography is better suited to confirming truly healthy tonsils and avoiding unnecessary surgery in equivocal cases than to reliably excluding disease. Given its relatively low sensitivity, elastography should not be used as a standalone screening tool or to replace clinical criteria but rather as a confirmatory adjunct within the existing diagnostic pathway. Serial SWE might also support the longitudinal follow-up of CT, for example, to monitor treatment response or progression toward fibrosis, whereas the lower-cost SR may be useful for screening in resource-limited settings despite its lower reproducibility. Overall, the SR and SWE may provide complementary information, with the SR serving as an initial screen and SWE as a confirmatory measure; this approach is especially suitable for children, for whom invasive assessment is undesirable.

A further consideration is the significant age differences between the groups. Patients with CT were older than those with ATH and controls. This partly reflects the natural epidemiology of the two conditions, as obstructive ATH predominates in preschool and early school-age children, whereas chronic or recurrent tonsillitis more often presents in older children and adolescents. However, because the palatine tonsil size and tissue composition change physiologically with age, increasing in the first years of life and gradually regressing thereafter [29], age may also influence elastographic stiffness independently of disease. As the CT group was the smallest and its members the oldest, its high stiffness values cannot be attributed to inflammation and fibrosis alone, and an age-related contribution cannot be excluded.

Several limitations should be acknowledged. The CT subgroup was small and its members significantly older than those in the other groups, which raises the possibility of an age effect on stiffness. The cross-sectional design could not capture treatment effects or temporal change, and no histopathological correlation was available. In addition, diagnostic performance was assessed for each elastography parameter individually. Given our limited and unbalanced sample size, particularly the small CT subgroup, we did not attempt to combine parameters using multivariate classifiers such as logistic regression or machine-learning-based methods (e.g., random forest), as these would be prone to overfitting in this cohort. Future multicenter studies with larger, age-matched cohorts; histopathological validation; and longitudinal follow-up are needed to refine diagnostic cut-offs and to determine whether elastographic stiffness can predict the clinical outcome or treatment response.

## 5. Conclusions

Both the SR and SWE proved to be useful objective tools for distinguishing normal tonsils from those affected by ATH and CT. SWE showed the highest diagnostic accuracy, and the submandibular gland was a more reliable reference tissue than the tongue for strain-based measurement. The higher SR and SWE values in diseased tonsils are consistent with the expected histopathological changes in lymphoid hyperplasia, fibrosis, and chronic inflammatory remodeling, supporting the biological relevance of elastographic stiffness. The strong bilateral correlations and the absence of sex-related differences further support the consistency of elastography in tonsillar assessment. As a non-invasive, real-time complement to traditional ultrasonography, SWE can aid in the diagnostic differentiation of chronic tonsillitis, treatment planning, follow-up, and the avoidance of unnecessary surgeries. Larger multicenter studies with histopathological validation are required to establish standardized cut-off values and to confirm the prognostic role of elastography in otolaryngologic practice.

## Figures and Tables

**Table 1:** Demographic characteristics of the study groups.

Variable	Adenotonsillar Hypertrophy (*n* = 55)	Chronic Tonsillitis (*n* = 10)	Control (*n* = 31)	*p*-Value
Age, Years, Median (IQR) [Min–Max]	8 (6–12) [3,4,5,6,7,8,9,10,11,12,13,14,15,16,17]	14 (11.5–15) [10,11,12,13,14,15,16,17]	8 (5–12) [3,4,5,6,7,8,9,10,11,12,13,14,15,16,17]	0.003 *
Female, *n* (%)	24 (43.6%)	6 (60.0%)	13 (41.9%)	0.587 †
Male, *n* (%)	31 (56.4%)	4 (40.0%)	18 (58.1%)	

* Kruskal–Wallis test, † Pearson’s chi-square test; χ^2^ = 1.067, df = 2.

**Table 2:** Comparison of tonsillar strain ratios and shear wave elastography measurements among adenotonsillar hypertrophy, chronic tonsillitis, and control groups.

Parameter	Adenotonsillar Hypertrophy (*n* = 55) Median (IQR)	Chronic Tonsillitis (*n* = 10) Median (IQR)	Control (*n* = 31) Median (IQR)	*p*-Value *
Right Tonsil–Tongue SR	1.43 (1.27–1.57)	5.73 (3.66–7.08)	1.29 (1.21–1.41)	<0.001
Left Tonsil–Tongue SR	1.43 (1.23–1.62)	4.16 (2.30–4.57)	1.35 (1.23–1.43)	<0.001
Right Tonsil–Submandibular Gland SR	3.20 (2.78–4.02)	4.03 (2.59–5.73)	2.32 (2.02–3.00)	<0.001
Left Tonsil–Submandibular Gland SR	3.31 (2.70–4.04)	5.28 (4.46–6.52)	2.22 (2.04–2.77)	<0.001
Right SWE (kPa)	6.40 (5.15–8.30)	12.65 (9.75–13.37)	3.80 (3.50–4.45)	<0.001
Left SWE (kPa)	6.10 (5.25–8.25)	10.50 (8.94–12.12)	4.10 (3.50–4.65)	<0.001

Abbreviations: SR, strain ratio; SWE, shear wave elastography; kPa, kilopascal. * *p*-value of <0.05 (statistically significant).

**Table 3:** Diagnostic performance of elastography parameters for differentiating tonsillar disease (ATH + CT) from healthy controls.

Parameter	AUC (95% CI)	Cut-Off	Sensitivity (95% CI)	Specificity (95% CI)
Right SWE (kPa)	0.918 (0.864–0.973)	5.15	0.785 (0.677–0.877)	0.968 (0.903–1.000)
Left SWE (kPa)	0.892 (0.830–0.955)	5.25	0.785 (0.692–0.877)	0.871 (0.742–0.968)
Left Tonsil–SMG SR	0.844 (0.767–0.920)	3.08	0.662 (0.538–0.769)	0.935 (0.839–1.000)
Right Tonsil–SMG SR	0.798 (0.706–0.889)	3.04	0.677 (0.569–0.785)	0.774 (0.613–0.903)
Right Tonsil–Tongue SR	0.727 (0.627–0.827)	1.42	0.585 (0.462–0.708)	0.839 (0.677–0.935)
Left Tonsil–Tongue SR	0.656 (0.546–0.766)	1.44	0.554 (0.431–0.677)	0.774 (0.613–0.903)

Abbreviations: AUC, area under the curve; CI, confidence interval; SMG, submandibular gland; SR, strain ratio; SWE, shear wave elastography.

## Data Availability

The original contributions presented in this study are included in the article. Further inquiries can be directed to the corresponding author.

## Abbreviations

The following abbreviations are used in this manuscript:

SR	strain ratio
SWE	shear wave elastography
ATH	adenotonsillar hypertrophy
CT	chronic tonsillitis
MRI	magnetic resonance imaging
